# Developing a Core Outcome Set for Prognostic Research in Palliative Cancer Care: Protocol for a Mixed Methods Study

**DOI:** 10.2196/49774

**Published:** 2023-09-01

**Authors:** Caitlin Spooner, Bella Vivat, Nicola White, Patrick Stone

**Affiliations:** 1 Marie Curie Palliative Care Research Department University College London London United Kingdom

**Keywords:** core outcome set, palliative care, end-of-life, prognosis, advanced cancer, systematic review, interviews, Delphi study

## Abstract

**Background:**

Studies exploring the impact of receiving end-of-life prognoses in patients with advanced cancer use a variety of different measures to evaluate the outcomes, and thus report often conflicting findings. The standardization of outcomes reported in studies of prognostication in palliative cancer care could enable uniform assessment and reporting, as well as intertrial comparisons. A core outcome set promotes consistency in outcome selection and reporting among studies within a particular population. We aim to develop a set of core outcomes to be used to measure the impact of end-of-life prognostication in palliative cancer care.

**Objective:**

This protocol outlines the proposed methodology to develop a core outcome set for measuring the impact of end-of-life prognostication in palliative cancer care.

**Methods:**

We will adopt a mixed methods approach consisting of 3 phases using methodology recommended by the Core Outcome Measure in Effectiveness Trials (COMET) initiative. In phase I, we will conduct a systematic review to identify existing outcomes that prognostic studies have previously used, so as to inform the development of items and domains for the proposed core outcome set. Phase II will consist of semistructured interviews with patients with advanced cancer who are receiving palliative care, informal caregivers, and clinicians, to explore their perceptions and experiences of end-of-life prognostication. Outcomes identified in the interviews will be combined with those found in existing literature and taken forward to phase III, a Delphi survey, in which we will ask patients, informal caregivers, clinicians, and relevant researchers to rate these outcomes until consensus is achieved as to which are considered to be the most important for inclusion in the core outcome set. The resulting, prioritized outcomes will be discussed in a consensus meeting to agree and endorse the final core outcome set.

**Results:**

Ethical approval was received for this study in September 2022. As of July 2023, we have completed and published the systematic review (phase I) and have started recruitment for phase II. Data analysis for phase II has not yet started. We expect to complete the study by October 2024.

**Conclusions:**

This protocol presents the stepwise approach that will be taken to develop a core outcome set for measuring the impact of end-of-life prognostication in palliative cancer care. The final core outcome set has the potential for translation into clinical practice, allowing for consistent evaluation of emerging prognostic algorithms and improving communication of end-of-life prognostication. This study will also potentially facilitate the design of future clinical trials of the impact of end-of-life prognostication in palliative care that are acceptable to key stakeholders.

**Trial Registration:**

Core Outcome Measures in Effectiveness Trials 2136; https://www.comet-initiative.org/Studies/Details/2136

**International Registered Report Identifier (IRRID):**

DERR1-10.2196/49774

## Introduction

### Background

Palliative care is a service provided to patients with life-limiting illnesses, such as advanced cancer, focusing on improving quality of life and symptom management through the early identification, assessment, and treatment of pain and other ailments [1,2]. It can also enable patients to understand their choices for treatment while providing physical, psychological, social, and spiritual support to patients and their families or informal caregivers alike.

Prognostication is broadly defined as the process of making predictions about the future, such as the likely outcome or course of a disease and the chance of recovery or recurrence. This research is specifically concerned with prognostication of survival, or end-of-life (EOL) prognostication. Prognostication of EOL is a vital component of patient management and decision-making in palliative care [3,4]. An accurate prognosis of the EOL allows patients and their families or informal caregivers adequate time to prepare, such as making financial plans and identifying their preferences for place of death [5]. Medically, an accurate prognosis can allow clinicians to identify appropriate treatment strategies based on individual patients’ prognostic factors and symptoms [4].

Currently, there is no gold standard for prognostication of EOL. Many prognostic methods rely on clinician-estimated prognosis [5]. However, clinicians’ survival predictions are often inaccurate, inconsistent, and overoptimistic [6,7]. Various prognostic tools have been validated for use in palliative cancer care, and they may provide better accuracy, consistency, or other benefits in comparison with clinicians’ predictions [5]. However, the impact of such prognostic algorithms has yet to be established, and inconsistency in outcome reporting among prognostic studies makes it difficult to compare results.

### Rationale for Core Outcome Set Development

The selection of appropriate primary and secondary outcome measures is an essential component of study design; a study is only as credible as its end points [8]. However, researchers may select outcomes that do not reflect meaningful end points for all stakeholders, namely patients and informal caregivers or clinicians [9,10]. Inconsistency in end points across a particular area of research can be a barrier to comparing study findings, reducing the feasibility of meta-analysis, and perpetuating outcome reporting biases [10]. Heterogeneity of outcomes has been noted in palliative care research. Previous studies have often focused on outcomes deemed important by experts, namely academics and clinicians, thereby overlooking patients’ and other stakeholders' opinions and experiences and hindering the translation of findings into clinical practice [11-14].

A core outcome set (COS) promotes consistency in outcome selection among studies within a particular population, such as palliative cancer care, by providing a consensus-based, agreed minimum set of clinically meaningful outcomes to be measured and reported in all clinical trials of a specific population [15]. The Core Outcome Measures in Effectiveness Trials (COMET) initiative provides guidance for COS developers planning to construct a COS [15]. First, it is recommended that outcomes in existing published and ongoing studies be identified. Interviews with patients, informal caregivers or family, and other stakeholders can then be performed to ascertain outcomes important to them and assist research teams in understanding why particular outcomes are important [16]. Next, a Delphi process is advised, where key stakeholders such as patients, informal caregivers, and those who design and use research rank all the outcomes identified in the previous 2 phases until consensus is reached on which ones are the most important. The result is an agreed set of core outcomes.

There is as yet no consensus on which outcomes should be used to assess the impact of EOL prognostication in palliative cancer care. This study will develop a COS to assess prognostic impact, which will assist in preparing for future impact studies.

### Scope of the COS

Palliative care can be provided to people living with a variety of disease types and chronic illnesses, including but not limited to cancer, cardiovascular diseases, chronic respiratory disease, diabetes, and acquired immunodeficiency syndrome [1]. This study will focus on palliative care in patients with a life-limiting, advanced, or progressive cancer to avoid confounding outcomes that might arise from including all such a wide range of illnesses. This will be the first study to investigate and define a COS for prognostic research in palliative cancer care. For the purpose of this study, the term *palliative cancer care* will be defined as treatment, care, and support provided to those with a life-limiting, advanced, or progressive cancer that cannot be cured [1,2]. This is a working definition, in that this definition has been chosen for the purpose of this study, and we recognize that it may not fully conform with established or authoritative definitions of *palliative cancer care*. Informal caregivers will include family members, friends, or other individuals who provide a wide range of unpaid assistance for someone with whom they have a personal relationship, including but not limited to physical and psychological support [17,18].

The intervention of interest in our study is EOL prognostication, defined as any process of estimating and communicating the likelihood of survival (ie, how long an individual has left to live) [5].

### Aim and Objectives

This study aims to create a COS for measuring the impact of EOL prognostication in palliative cancer care.

The objectives for the whole study are as follows:

1. To develop a list of outcomes relevant to the impact of EOL prognostication in palliative cancer care.
2. To prioritize outcomes for measuring the impact of EOL prognostication in palliative cancer care that are important to relevant stakeholders, namely, patients, informal caregivers, and clinicians.
3. To achieve consensus on a minimum set of relevant outcomes to assess the impact of EOL prognostication in palliative cancer care.

## Methods

### Design

This study will adopt a multiphase, mixed methods approach suggested by the COMET guidelines [15] and will adhere to the Core Outcome Set—STAndards for Development (COS-STAD) recommendations to support the development, implementation, and evaluation of the COS [19]. This study protocol was written following the Core Outcome Set—STAndardized Protocol Items (COS-STAP) guidelines for reporting protocols of COS development [20].

**Figure 1:**
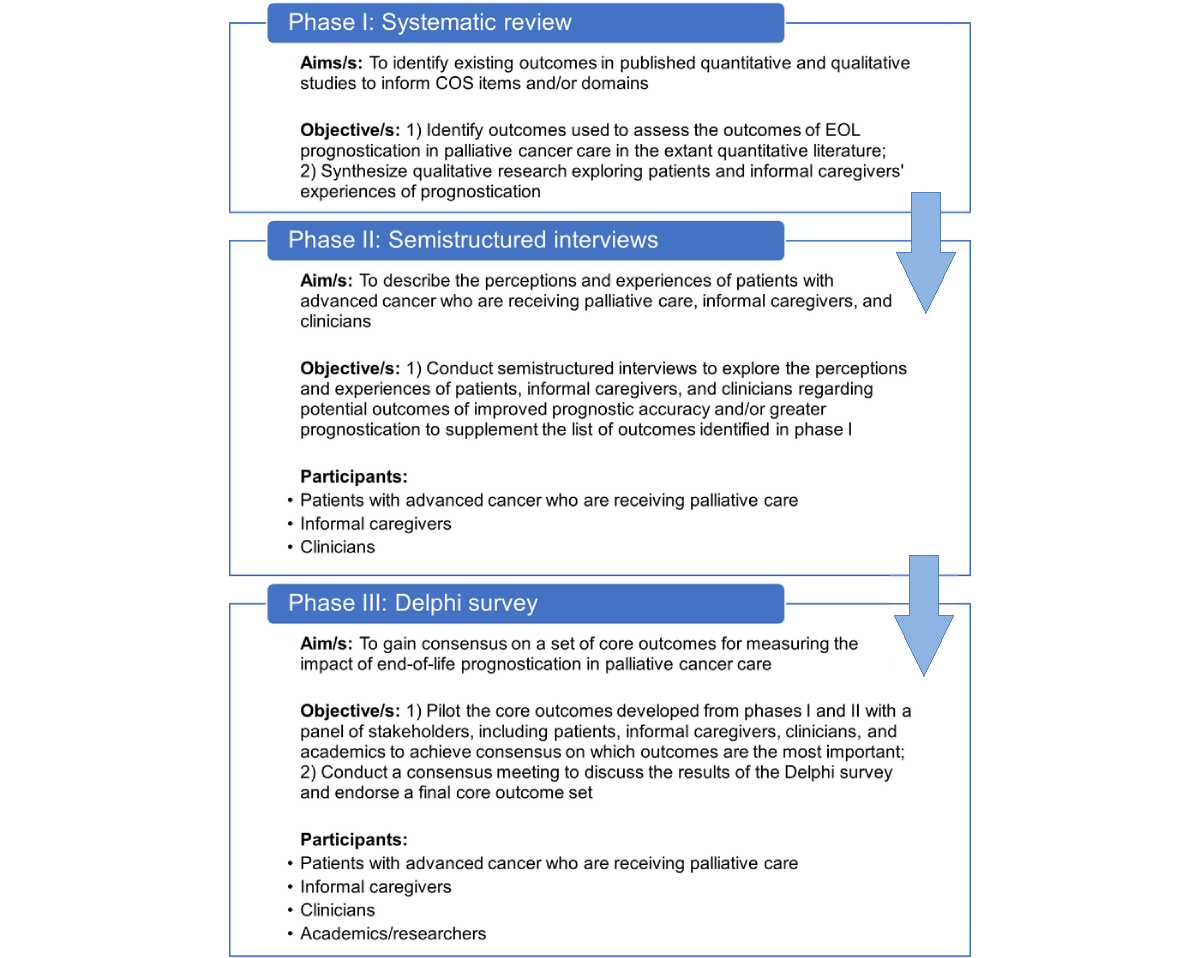
Phases of developing a core outcome set (COS) for the impact of end-of-life (EOL) prognostication in palliative cancer care.

The study will consist of three phases: (1) a systematic literature review of relevant outcomes reported in extant quantitative and qualitative literature to measure the impact of EOL prognostication in palliative cancer care; (2) semistructured, qualitative interviews to explore the views and experiences of patients, informal caregivers, and clinicians; and (3) a Delphi study to prioritize outcomes derived from phases I and II. Figure 1 shows a summary of the study design.

### Stakeholders and Setting

This study will involve four stakeholder groups: (1) patients with advanced cancer who are receiving palliative care, (2) informal caregivers, (3) clinicians, and (4) academics or researchers. We will recruit patients, informal caregivers, and clinicians through palliative care services from 3 sites in London, United Kingdom, including 2 National Health Service trusts and 1 hospice. We will begin recruitment to the study by placing posters advertising the study in public spaces of the participating sites to recruit patients (both inpatients and outpatients), informal caregivers, and clinicians simultaneously. These posters will include the researchers’ contact information so that interested individuals can contact them for further details regarding the study, to ask questions, or to express their interest in participating.

Where possible, a researcher (CS) will also attend weekly multidisciplinary team meetings within the sites and be available on site to encourage clinical staff to identify patients who are eligible to participate and who are physically and mentally capable. The researcher will only attend multidisciplinary team meetings at a time point agreed with the clinical team (ie, at the beginning or the end) to discuss the study and will leave before any confidential patient information is discussed. A member of the patient’s direct clinical care team (ie, someone who is currently treating them) will make the first approach to potential participants to ask them if they would be interested in participating in the study, and if so, whether they give permission for the clinical team to provide the minimum, necessary information (name and contact details) for the researcher to approach them. The researcher will only approach participants who have consented (ie, verbally) for them to do so. The researcher or clinical staff will provide such patients with a participant information sheet and the researchers’ contact information. If present at the time, informal caregivers will also be asked if they would be interested in participating in the study.

An invitation email, participant information sheet, and researcher contact information will be shared with palliative care clinicians at participating sites. We will also contact relevant organizations, such as the Association for Palliative Medicine of Great Britain and Ireland and the European Association for Palliative Care, to recruit national and international palliative care clinicians. We will ask the group or association chair to forward an email to group members that contains the participant information sheet and a link to the first round of the survey.

We will identify academics or researchers through opportunity sampling. We will ask colleagues and other contacts to suggest potential participants who have the relevant experience and are able to read and write in English. To encourage participation from a wide geographical area, we will screen academic profiles on UK and international university websites to identify individuals who have conducted relevant work. Heads of palliative care research departments may also be contacted and asked whether any members of their team have conducted research within the field of palliative care and would be willing to put us in contact. Additionally, the reference list of any relevant reviews will be searched to identify academics who have published research in the palliative care settings, and the authors who have cited relevant papers will also be screened for eligibility.

For phase II, we estimate that we will need to recruit 8 to 10 participants per group (patients, informal caregivers, and clinicians), making a total of 24 to 30 participants. This sample size is based on a previous COS study’s recommendations, as well as the research team’s experiences of qualitative research [21]. However, data collection will continue until data saturation is reached. Data saturation will be determined to have occurred when no new themes are being identified in analysis. Data collection will occur concurrently with recruitment to minimize attrition.

There is currently no consensus on how to determine the sample size for a Delphi survey. Balancing the sample size in a Delphi survey is crucial for achieving meaningful consensus; larger sample sizes may present challenges in terms of communication and follow-up, leading to lower response rates, whereas smaller sample sizes can restrict the scope and reliability of the survey results [22-24]. When considering sample size, it is also important to consider attrition that is likely to occur between each round. Therefore, based on previous studies’ recommendations [25,26], we took a pragmatic decision to recruit a minimum of 10 participants to represent each stakeholder group, with a minimum of 40 participants in total. For pragmatic reasons, and to ensure that we include a range of perspectives, we aim to recruit a minimum of 10 international experts. For the consensus meeting, we will recruit approximately 4 participants from each stakeholder group. Maintaining a balance between stakeholder groups is important to ensure that certain voices are not overpowered. This group size will be manageable for a web-based meeting, while still permitting a range of perspectives to be heard.

### Phase I: Systematic Literature Review

Phase I of the study was completed in June 2023. The complete protocol for this systematic literature review, including the search strategy and study selection criteria, was submitted for registration on PROSPERO (the International Prospective Register of Systematic Reviews) on March 29, 2022, and was subsequently published on the PROSPERO website on April 1, 2022 (CRD42022320117). This review was conducted according to the PRISMA (Preferred Reporting Items for Systematic Reviews and Meta-Analyses) 2020 statement [27].

The aim of this systematic literature review was to identify existing outcomes to inform COS items and domains. Retrieved papers were analyzed in two subsets: (1) quantitative studies (clinical trials and observational studies), seeking to identify the outcomes used in previous research to measure the impact of EOL prognostication in palliative cancer care, and (2) a systematic review and thematic synthesis of qualitative studies exploring patients’ and informal caregivers’ experiences of EOL prognostication in palliative cancer care.

We carried out searches using the following electronic databases from inception to September 2022: Ovid MEDLINE, Ovid Embase, Ovid PsycINFO, CINAHL, and the Cochrane Library (both the Cochrane Controlled Register of Trials and the Central Register). We also extended searches to gray sources and adopted snowballing techniques to identify additional papers and ensure literature saturation. Search terms included keywords related to prognostication, palliative care, advanced cancer, and outcomes and cognate terms. Search limits were applied to restrict results to a human, adult population, and studies published in English language only due to limitations in resources. Eligibility criteria included (1) patients with advanced cancer, or their informal caregivers; (2) prognostication, defined as any process of estimating and communicating the length of survival of an individual’s disease; (3) quantitative studies that reported outcomes of prognostication; and (4) qualitative studies that explored experiences and perceptions of prognostication.

The search results from each database were imported and deduplicated in the Rayyan software [28]. The titles and abstracts of the studies were screened by 4 independent reviewers using the above-mentioned eligibility criteria. Those that met the inclusion criteria for either review question were read in full by at least 2 authors. Discrepancies were resolved through discussion between authors. If no consensus could be reached, a third author was consulted.

Methodological quality was assessed using the Mixed Methods Appraisal Tool [29]. A narrative approach was used to undertake the quantitative synthesis, along with tables of outcomes, their descriptions, and measures. Outcomes were categorized using the taxonomy for COS recommended by COMET [30]. For the qualitative analysis, the analysis was guided by Thomas and ’Harden’s [31] framework for thematic synthesis, with 3 stages. The COMET taxonomy was used as an external framework to support the development of analytical themes and facilitate the combination of the results of both the quantitative and qualitative studies. A narrative description of each theme in relation to the review question was provided.

The results of the review have been accepted for publication in an academic journal and will be taken forward for consideration for inclusion in the COS.

### Phase II: Semistructured Interviews

The second phase of this study will be a qualitative study using semistructured interviews to explore the perceptions and experiences of patients, informal caregivers, and clinicians regarding the potential outcomes and impact of EOL prognostication.

#### Participants

We will use purposive, maximum variation sampling to capture a range of patients, informal caregivers, and clinicians with the characteristics shown in Textbox 1. To achieve this, we will collaborate closely with staff at participating sites and seek to identify a diverse sample, accounting for characteristics such as age, gender, ethnicity, socioeconomic status, education level, cancer diagnosis, relationship to the person with cancer, and professional role. This will enable us to ensure that our study includes a broad spectrum of perspectives and experiences, making our research findings more comprehensive and representative. For pragmatic reasons related to resource availability, we will only be able to recruit participants who are able to communicate in and understand written and spoken English.

**Eligibility criteria for phase II.**

**Inclusion criteria**

- Patients
  Aged ≥18 years
  Receiving palliative cancer care as per the prespecified definition [1,2]
- Informal caregivers
  Aged ≥18 years
  Identifies as an informal caregiver, as per the prespecified definition [17,18]
  Have provided such care within the last 12 months before study recruitment, irrespective of survival (in cases where the informal caregiver is bereaved, they will be contacted no less than 6 months after bereavement out of respect and concern) [32]
- Clinicians
  A registered health care professional (eg, a physician, nurse, or allied health professional) employed in the palliative care services at one of the participating sites, who routinely estimates and provides end-of-life (EOL) prognostic predictions

**Exclusion criteria**

- Patients
  Children (aged <18 years)
  Individuals who, in the opinion of their treating clinician, would not be appropriate to participate in this research
  Individuals who have previously expressed a wish not to be involved in research
  Individuals who lack capacity to give informed consent
- Informal caregivers
  Young informal caregivers (aged <18 years)
  Informal caregivers who, in the opinion of the clinician gatekeeper, would be inappropriate to approach about enrollment in the study due to physical or mental illness
  Individuals who lack capacity to give informed consent
- Clinicians
  Clinicians whose role in palliative care is undertaken exclusively outside of the United Kingdom (to avoid varying, potentially confounding practices)
  Clinicians who do not routinely estimate and provide EOL prognostic predictions in palliative cancer care

#### Data Collection

Participants will be invited to take part in one semistructured interview, which will be conducted by one researcher (CS) with individuals either face-to-face, via video call, or via telephone, depending on practical considerations, participant preferences, and any prevailing COVID-19 restrictions. Interviews are anticipated to last between 30 minutes and 1 hour and will be conducted in a quiet environment, at a convenient time for the participant. From our experience, people with advanced cancer may only be willing or able to be interviewed for 30 minutes at a time, so we will be flexible in meeting their needs and will either continue for longer if they are comfortable or take a break. Before the interview starts, the interviewer will explain the concept of the COS and the importance of measuring the impact of prognostication in palliative care. Participants will also be asked to provide basic demographic information for descriptive reasons only.

The interviews will be semistructured using a topic guide. The main topics covered will be consistent across the interviews, but the specific phrasing of questions and when they are posed during the interview will vary depending on how the conversation develops throughout the interview. Interviews will be audio-recorded with the participant’s consent.

#### Data Analysis

Interview transcripts will be analyzed thematically using a framework analysis approach. Audio recordings will be transcribed verbatim. A researcher (CS) will independently read the transcripts and generate initial codes. A second researcher (BV) will independently read a sample set of interviews to provide a second, independent perspective and ensure that all codes capture the range and depth of data. The 2 researchers will develop initial proposals for themes and then meet to discuss and agree broad themes. CS will then review and construct a final set of themes. The data from these interviews will be analyzed alongside the data from phase I, so as to identify outcome domains to be included in the long list of core outcomes to be taken forward into phase III. Descriptive statistics will be used to present demographic information.

### Phase III: Delphi Survey

Once a list of core outcomes has been developed from phases I and II, we will carry out a Delphi survey and consensus meeting to achieve consensus between disparate stakeholders, including patients, informal caregivers, clinicians, and academics or researchers, about which of these outcomes are most important. An international sample of clinicians and academics or researchers will be sought to widen the generalizability of the final COS.

#### Participants

The goal is to have representation of different perspectives about which are the important outcomes to include in the COS. The Delphi panel members should reflect the population that is intended to use the COS or who will be involved in subsequent research studies that use the COS [33]. Therefore, the participant panel will comprise 4 key stakeholder groups: patients, informal caregivers, clinicians with clinical experience in palliative care, and academics or researchers with research experience in palliative care. We will use convenience sampling, as well as opportunity and snowball sampling to achieve a maximum variation sample by including people with the characteristics shown in Textbox 2.

**Eligibility criteria for phase III.**

**Inclusion criteria**

- Patients
  Aged ≥18 years
  Receiving palliative cancer care as per the prespecified definition [1,2]
- Informal caregivers
  Aged ≥18 years
  Identifies as an informal caregiver, as per the prespecified definition [17,18]
  Have provided such care within the last 12 months before study recruitment, irrespective of survival (in cases where the informal caregiver is bereaved, they will be contacted no less than 6 months after bereavement out of respect and concern) [32]
- Clinicians
  A registered health professional (eg, a physician, nurse, or allied health professional) with experience in estimating and providing end-of-life (EOL) prognostic predictions in palliative care and who is currently or was recently clinically employed (ie, not retired for >12 months or dismissed from clinical work)
  Palliative care experience can be within the United Kingdom or international
- Academics or researchers
  Academics or researchers who have published research, written or supervised a thesis, or contributed to a book chapter on the topic of prognostication in palliative care
  Research experience can be within the United Kingdom or international
  These experts may also be clinicians; however, this will not be a requirement

**Exclusion criteria**

- Patients
  Children (aged <18 years)
  Individuals who, in the opinion of their treating clinician, would not be appropriate to participate in this research
  Individuals who have previously expressed a wish not to be involved in research
  Individuals who lack capacity to give informed consent
- Informal caregivers
  Young informal caregivers (aged <18 years)
  Informal caregivers who, in the opinion of the clinician gatekeeper, would be inappropriate to approach about enrollment in the study due to physical or mental illness
  Individuals who lack capacity to give informed consent
- Clinicians
  Clinicians who are not clinically employed in the last 12 months (ie, retired or dismissed from clinical work)
  Clinicians who do not routinely estimate and provide EOL prognostic predictions in palliative cancer care
- Academics or researchers
  Academics or researchers whose experience lies within the generic topic of palliative care without any specific experience in prognostication

#### Data Collection

The Delphi survey will comprise multiple web-based surveys that will be answered anonymously by stakeholders until consensus is achieved about which outcomes should be included in the COS. The outcomes resulting from the systematic review and the semistructured interviews will be prioritized by the participants in up to 3 of these web-based Delphi rounds via secure, web-based software (REDCap; Vanderbilt University) [34,35].

In round 1, participants will be asked to grade the importance of each outcome on the long list relative to the other outcomes in terms of how important they think they are in measuring the impact of improved prognostication. A 9-point Likert scoring system will be used, where a score of 1 to 3 denotes “low importance,” 4 to 6 is “important, but not critical,” and 7 to 9 is “critical,” as per the GRADE (Grading of Recommendations Assessment, Development and Evaluation) scale [36]. Participants will also be provided with an “unable to score” response if they consider themselves unable to rate any of the outcomes, as well as a free textbox to provide any comments.

It is anticipated that each survey will take approximately 30 minutes to complete. Participants will be given 2 weeks to respond to the survey. Participants will also have the opportunity to identify any outcomes that they think are relevant from their perspective, but which were not included in the initial list. This will facilitate the inclusion of outcomes that may be relevant but were not obtained in phases I and II of the study.

The group’s ratings will be analyzed together, and outcomes removed or included depending on the group consensus. The survey will be repeated (with modifications) up to 3 times. Group descriptive statistics and a summary of comments will be generated after each round and sent to the participants during the next round of the survey. All comments and ratings shared will be anonymous.

**Figure 2:**
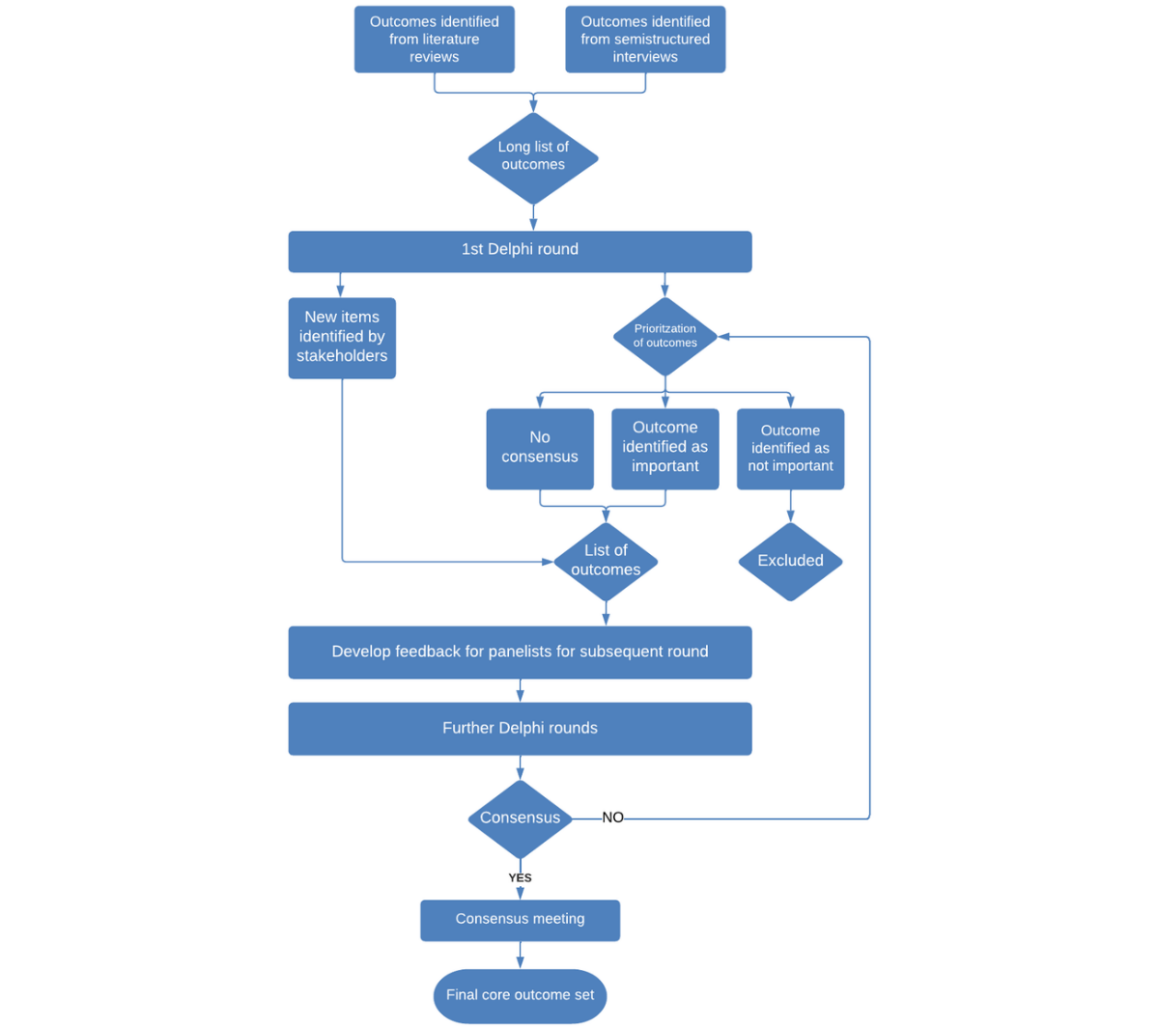
Flow chart of the Delphi process.

If after 3 rounds, items remain without consensus, the Delphi survey will be terminated and the outcomes with no consensus, alongside those with consensus for inclusion and exclusion, will be presented at the consensus meeting where a final decision will be made. The aim of this meeting will be to discuss the results of the Delphi survey and vote on the final COS. Participants for the consensus meeting will be purposefully selected from the Delphi survey participants. The consensus meeting will be conducted via a web platform for practical reasons, allowing for the inclusion of international participants and accommodating any prevailing COVID-19 restrictions. This remote format is generally adopted in COS consensus meetings as it ensures accessibility, safety, and global representation, promoting a more inclusive and fruitful discussion during the meeting [37]. The panel will be asked to confirm whether they agree (or not) with the inclusion or exclusion of the outcomes in the COS. The result of this will be the final, consensus-agreed COS. Figure 2 summarizes the Delphi process proposed in this study.

#### Data Analysis

To determine which items should be included in the final COS, the thresholds recommended by the COMET initiative will be used [10]. Consensus for inclusion or “consensus in” will be defined as ≥70% of all participants rating the outcome as “critical” (GRADE score 7-9) and ≤15% of all participants rating the outcome as of “low importance” (GRADE score of 1-3). Consensus for exclusion or “consensus out” will be defined as ≥70% of the participants rating the outcome as of “low importance” and ≤15% of all responses rating the outcome as “critical.” Outcomes meeting consensus for “not important for inclusion” will be removed and the remaining outcomes carried forward to the next round. Outcomes that are rated as “critical” or of “low importance” by one panel will be carried forward to the next Delphi round. For example, outcomes rated <70% by informal caregivers, clinicians, and academics, but with a patient rating of >70%, will be considered “patient-important” and included in the next round.

The same definitions for “consensus in” or “consensus out” will be applied to the consensus meeting.

### Patient and Public Involvement

We advertised the opportunity to be involved in this research project as part of a patient and public involvement (PPI) advisory group through the National Institute for Health Research “People in Research” website and the Marie Curie Research Voices Group. Our advisory group included members of the public with experience of talking about prognosis, either as someone with advanced cancer or as one caring for someone with advanced cancer, who reviewed the protocol and all participant-facing documents. They advised on how the protocol could be adapted to ensure that the research study meets the needs and preferences of those taking part. They also made suggestions for how the documents might be made more suitable for participants, particularly for those receiving palliative care, to ensure that sensitive language was used.

In the future, we will consult this PPI advisory group regarding the format of the Delphi survey, before distributing it to participants, to ensure that it is easy to understand, access, and use. Specifically, the group will be requested to review and streamline the extensive list of outcomes intended for inclusion in the Delphi survey. We recognize that lengthy lists can be daunting for Delphi participants, potentially impacting their engagement. The PPI advisory group’s expertise will be invaluable in identifying and addressing this risk, guiding us in minimizing the occurrence of overly lengthy lists and ensuring that the Delphi survey is well received and productive. We will also ask the group to review and propose outcome names and plain-language descriptions for each outcome. This step aims to ensure that all participants interpret the concepts as intended, reducing the potential for misunderstandings and enhancing the overall clarity of the survey.

During the data analysis, we will ask members to comment on the plain-language presentation of findings to ensure that this is understandable and relevant. We will also seek input from members of the PPI advisory group when disseminating results of the study to ensure that we communicate the study in a way that is appropriate for the general public.

The involvement of the PPI advisory group in these crucial aspects of the study has already made, and will continue to make, a considerable contribution to the success and impact of our research. In particular, this facilitates more meaningful participation and the likelihood of gaining valuable insights from participants.

### Ethics Approval and Informed Consent

Ethical approval was obtained for this study from the London-Camberwell St. Giles Research Ethics Committee and the Health Research Authority on September 6, 2022 (reference 22/LO/0469).

Written, informed consent will be obtained from participants taking part in face-to-face interviews, the first round of the Delphi survey, and consensus meeting. Verbal, recorded consent will be obtained from participants being interviewed over the telephone or via a web platform. Participation in sequential rounds of the Delphi survey will be considered indicative of consent.

### Dissemination

Promoting the uptake of the COS is paramount in preventing research waste and involves engaging key stakeholders, raising awareness, and demonstrating its value and relevance [38]. To achieve this, we will aim to disseminate findings to academic and research community audiences via publications in peer-reviewed journals and as presentations or posters at regional, national, and international palliative care conferences. After the finalization of the COS, further research will be necessary to identify the most suitable outcome measures or instruments for each core outcome. Subsequently, we will conduct a comprehensive evaluation of their quality and feasibility. To encourage adoption, we will collaborate with relevant journals and funding bodies, urging them to incorporate the COS in their guidelines for researchers and authors. Furthermore, we will actively seek feedback from researchers who pilot the COS in their studies. Being open to updates and revisions based on emerging evidence and feedback, we will continually improve the COS, ensuring its continued relevance and value over time.

The findings will be disseminated to participants in the form of plain-language summaries at the end of the study. To reach wider public audiences, plain-language newsletters, tweets, or blog posts will be published. Professional and clinical groups will also receive the findings as research summaries for participants and by engaging with palliative care representatives such as the palliative care team at the participating sites.

The final COS will be shared with the expert panel so they can verify the changes made and review what the project produced.

## Results

As of July 2023, we have completed and published the systematic review (phase I) and have started recruitment for phase II. So far, we have recruited 5 patients, 10 informal caregivers, and 10 clinicians. We are still recruiting patients for phase II. Data analysis for phase II has not yet started. We anticipate that the recruitment for the Delphi survey will begin in January 2024. We expect to complete the study by October 2024 and publish the results by June 2025.

## Discussion

### Anticipated Study Findings and Potential Impact

This study protocol presents what will be the first study that aims to develop a COS to measure the impact of EOL prognostication in palliative cancer care. The objective of this COS is to involve various stakeholders in its development and reach a consensus on the essential outcomes that hold significance for this population. Developing a COS for measuring the impact of EOL prognostication in palliative cancer care is an important step to improve patient care and communication of EOL prognoses. By addressing the inconsistent outcome measurement in existing studies and aligning the outcomes with those that are important to stakeholders, our COS has the potential to help standardize and improve prognostic research in palliative cancer care, such as the development of prognostic algorithms. Furthermore, the findings from this study have the potential to ensure that outcomes measured in future prognostic impact studies will reflect what is important to such stakeholders and ultimately facilitate the translation of the findings into clinical practice [39-41]. Once the COS is finalized, further research will be required to establish which outcome measures or instruments are most appropriate for each of the core outcomes, followed by an evaluation of their quality and feasibility.

### Comparison With Existing Literature

Currently, research on the impact of EOL prognostication in palliative cancer care faces a significant challenge because of the measurement of different outcomes across various studies. Academic and clinical interests often dominate the selection of outcomes, potentially neglecting their meaningfulness to patients or informal caregivers [9,10]. Consequently, some studies might employ outcomes that do not resonate with those directly impacted by prognostic information. Moreover, the lack of uniformity in outcome measurement across studies hampers the ability to compare or combine results, leading to research wastage. A COS will address these issues. This COS aims to integrate the perspectives and experiences of patients and other personally affected stakeholders. By doing so, it is expected to enhance the translation of research findings into clinical practice and foster comparability in future prognostic research in palliative cancer care. Ultimately, this harmonized approach will optimize the use of research efforts and promote more effective advancements in the field.

### Strengths and Limitations

This will be the first COS specifically focused on measuring the impact of EOL prognostication in palliative cancer care. The development of the COS will involve a rigorous and systematic search to gather the views and opinions of patients and other key stakeholders. Engaging with these groups will not only ensure that their perspectives are considered but will also enhance the overall relevance and applicability of the COS. By making the protocol for the COS publicly available, we will ensure the transparency and replicability of our study.

The inclusion of only adult English-speaking participants in this study might potentially limit the generalizability of the findings. Nevertheless, to mitigate this limitation, we will actively seek the involvement of international participants in the Delphi survey. Additionally, in phase I of the study, the systematic review considered international publications to minimize the risk of selection bias.

It is essential to acknowledge that conducting the Delphi survey using a web-based software may lead to underrepresentation of certain groups, particularly those who lack digital skills and access. To address this concern, we will explore alternative methods of data collection to ensure broader representation if it becomes apparent that our sample is significantly biased.

Recruiting and retaining participants may pose a challenge in our study, especially given the nature of the target population, who may face health deterioration or bereavement. To minimize attrition during phase II, we will conduct interviews concurrently with recruitment, aiming to interview participants shortly after enrollment to reduce dropouts. If a participant or caregiver cannot proceed with the interview, we will document their reasons for withdrawal and continue recruiting until reaching our desired number of interview participants or research data saturation, whichever occurs first. Attrition in Delphi surveys is a common challenge, resulting from both participant factors and reduced interest over time during multiple rounds [42]. Although we will send regular reminders and strive for timely rounds, it is challenging to minimize attrition resulting from external circumstances. Furthermore, the survey’s anonymity prevents us from knowing specific reasons for dropouts or withdrawals. Despite this limitation, we will openly acknowledge its impact in any published results.

Employing stakeholder meetings to determine the final COS carries the risk of bias based on the opinions expressed by those present. To counteract this, we will make diligent efforts to encourage participation from all stakeholder groups. Despite our efforts, there remains a possibility of underrepresentation for a particular stakeholder group, and we will be transparent about this potential limitation in our study.

### Conclusions

The necessity for a COS to measure the impact of EOL prognostication within palliative cancer care arises from the need to enhance outcome measurement and reporting. This COS has the potential to make a substantial impact on the field by advancing prognostication practices and aligning outcome measures with the preferences and requirements of patients and other important stakeholders. As a result, it will enable more precise comparisons and better synthesis of evidence across various prognostic studies.

The overall promise of this study lies in its potential to elevate the quality of care and decision-making for patients facing EOL care in the context of palliative cancer care. By standardizing and improving the measurement and reporting of prognostic outcomes, the COS will play a vital role in optimizing patient outcomes and enhancing the overall care experience for those in need.

## Abbreviations

COMET: Core Outcome Measures in Effectiveness Trials
COS: core outcome set
COS-STAD: Core Outcome Set—STAndards for Development
COS-STAP: Core Outcome Set—STAndardized Protocol Items
EOL: end of life
GRADE: Grading of Recommendations Assessment, Development and Evaluation
PPI: patient and public involvement
PRISMA: Preferred Reporting Items for Systematic Reviews and Meta-Analyses
PROSPERO: The International Prospective Register of Systematic Reviews

## Data Availability

The data sets generated during and/or analyzed during this study are not publicly available due to the sensitive nature of the information, and participants’ consents for public data sharing were not obtained in this study.
